# Development of core outcome set for pediatric health conditions: a systematic review

**DOI:** 10.1186/1745-6215-16-S1-P25

**Published:** 2015-05-29

**Authors:** Mufiza Kapadia, Winnie Chan, Thivia Jegathesan, Martin Offringa

**Affiliations:** 1 Child Health Evaluation Sciences, The Hospital for Sick Children, Toronto, Canada; 2 Department of Paediatrics, University of Toronto, Toronto, Canada

## Background

Standardized selection of outcomes has been advocated to allow comparison and syntheses of clinical trials’ results in systematic reviews and to avoid outcome selection bias. As health outcomes in children are different from adults, the methodology behind selecting and measuring outcomes should be valid, responsive and feasible for pediatric research. Core Outcome Sets (COS) are a new opportunity to attain these criteria.

## Objectives

Our aim is to synthesise evidence on methods used for “developing” and “validating” COS in pediatric studies, and to determine what domains (common as well as specific) are identified within the studies.

## Methods

We searched Medline, EMBASE, COMET and PsychInfo for relevant articles in the English language from their inception to June 2014. COS developed for paediatric health conditions (aged <18 years) and published in English language are included. Titles and abstracts are screened for inclusion by two reviewers. The reference list of the included articles are screened for further studies. Outcomes included within COS are mapped into five core areas of OMERACT filter 2.0, namely pathophysiological manifestations, adverse events, life impact, resource use and death.

## Results

The literature search identified 7,100 non-duplicate abstracts. Of these, 72 full text articles are retrieved and 39 studies are included in this systematic review. Eleven studies focused on pediatric rheumatic diseases. 39% studies focused on paediatric rheumatic diseases. 38% studies employed a formal consensus technique (Delphi survey, nominal group technique or a questionnaire survey) to develop a COS. 21% studies undertook validation of the proposed COS. Only 10% reported involvement of parents or proxy in the consensus process. Apart from OMERACT filter 2.0 core areas, growth and development is another area identified in paediatric COS.

## Conclusion

Published experience showcasing the methodology of development and validation of COS in pediatric clinical research is limited. Methods used for outcome selection vary widely. Most reports are in pediatric rheumatic conditions. Development of standardised methodology for outcome selection and validation is recommended.

